# Coronavirus pandemic in the South Asia region: Health policy and economy trade-off

**DOI:** 10.7189/jogh.13.06014

**Published:** 2023-05-05

**Authors:** Furqan B Irfan, Ben Telford, Nick Hollon, Ali Dehghani, Casey Schukow, Ayesha Yasmeen Syed, Ryan T Rego, Akbar K Waljee, William Cunningham, Fahad Shabbir Ahmed

**Affiliations:** 1 Institute of Global Health, Michigan State University, East Lansing, Michigan, USA; 2 Department of Neurology and Ophthalmology, College of Osteopathic Medicine, Michigan State University, East Lansing, Michigan, USA; 3 College of Osteopathic Medicine, Michigan State University, East Lansing, Michigan, USA; 4 Wayne Medical Center, Westland, Michigan, USA; 5 Center for Global Health Equity, University of Michigan, Ann Arbor, Michigan, USA; 6 Department of Learning Health Sciences, University of Michigan, Ann Arbor, Michigan, USA; 7 Michigan Integrated Center for Health Analytics and Medical Prediction (MiCHAMP), University of Michigan, Ann Arbor, Michigan, USA; 8 Department of Pathology, Wayne State University, Detroit, Michigan, USA

## Abstract

**Background:**

The South Asian Association for Regional Cooperation (SAARC) covers Afghanistan, Bangladesh, Bhutan, India, Maldives, Nepal, Pakistan, and Sri Lanka. We conducted a comparative analysis of the trade-off between the health policies for the prevention of COVID-19 spread and the impact of these policies on the economies and livelihoods of the South Asia populations.

**Methods:**

We analyzed COVID-19 data on epidemiology, public health and health policy, health system capacity, and macroeconomic indicators from January 2020 to March 2021 to determine temporal trends by conducting joinpoint regression analysis using average weekly percent change (AWPC).

**Results:**

Bangladesh had the highest statistically significant AWPC for new COVID-19 cases (17.0; 95% CI = 7.7-27.1, *P* < 0.001), followed by the Maldives (12.9; 95% CI = 5.3-21.0, *P* < 0.001) and India (10.0; 95% CI = 8.4-11.5, *P* < 0.001). The AWPC for COVID-19 deaths was significant for India (6.5; 95% CI = 4.3-8.9, *P* < 0.001) and Bangladesh (6.1; 95% CI = 3.7-8.5, *P* < 0.001). Nepal (55.79%), and India (34.91%) had the second- and third-highest increase in unemployment, while Afghanistan (6.83%) and Pakistan (16.83%) had the lowest. The rate of change of real GDP had the highest decrease for Maldives (557.51%), and India (297.03%); Pakistan (46.46%) and Bangladesh (70.80%), however, had the lowest decrease. The government response stringency index for Pakistan had a see-saw pattern with a sharp decline followed by an increase in the government health policy restrictions that approximated the test-positivity trend.

**Conclusions:**

Unlike developed economies, the South Asian developing countries experienced a trade-off between health policy and their economies during the COVID-19 pandemic. South Asian countries (Nepal and India), with extended periods of lockdowns and a mismatch between temporal trends of government response stringency index and the test-positivity or disease incidence, had higher adverse economic effects, unemployment, and burden of COVID-19. Pakistan demonstrated targeted lockdowns with a rapid see-saw pattern of government health policy response that approximated the test-positivity trend and resulted in lesser adverse economic effects, unemployment, and burden of COVID-19.

The South Asian countries, comprising Afghanistan, Bangladesh, Bhutan, India, Maldives, Nepal, Pakistan, and Sri Lanka, account for 25% of the world population, but only 5% of the global gross domestic product (GDP) [1]. Their shared socio-political and economic challenges pushed them to form the South Asian Association for Regional Cooperation (SAARC) in 1985 [2]. Although Afghanistan was the last to join the SAARC in 2007, these countries have continued promoting their collective self-reliance and active collaboration with each other and other regional/international entities through summits [3]. Cooperation among neighbouring countries would be crucial in a successful response to a global health crisis, such as the COVID-19 outbreak which was declared a pandemic by the World Health Organization (WHO) on 11 March 2020 [4].

The SAARC nations are not new to epidemics of infectious diseases. However, the 2019 Global Health Security (GHS) index ranked the SAARC nations as “inadequately prepared” for a pandemic (with Afghanistan being the least and India being the most prepared) [5]. One reason was that these countries have limited critical care capacity, with approximately 0.7-2.8 critical care beds per 100 000 people, thus being unable to provide appropriate patient isolation and respiratory support in circumstances when outbreak containment is a top priority [6].

The SAARC countries faced the COVID-19 pandemic early on, initiating interventions (e.g. an emergency fund) as soon as February and March 2020 to limit its spread [7]. They also established containment measures such as restricting entry for incoming foreign nationals, suspending air travel, and contact tracing [8]. To prevent transmission and overburdening the health care system and to build public health capacity to contain the pandemic, most SAARC countries initiated complete lockdown measures [8]. As of 2013, these countries make up approximately 33.4% of the planet's low-income population. From the beginning of the COVID-19 pandemic, it was unclear how their economies and populations would cope with a complete lockdown [9].

Between an unstable health care system and rampant poverty, lockdown measures caused the GDP growth forecast to drop from 6.3% to 1.8%-2.8% [10,11]. Given the difficulty of maintaining physical distancing, lack of access to water in many areas, and poor health management systems, lockdown measures could not be kept in place for too long without adverse effects. The leaders of the SAARC countries had to balance between pandemic response measures (which could potentially lead to deaths due to poverty exacerbated by lockdowns and slowed economic activity) and downplaying the severity of the pandemic (which could cause many COVID-19-related deaths). Therefore, the South Asia region is useful for understanding how balancing pandemic response and public health measures helps save individuals from a highly contagious virus and reduce suffering and death related to economic collapse. We aimed to determine the COVID-19 health policies, epidemiology, and macroeconomic indicators of the SAARC countries to see how effective have these policies been in mitigating the pandemic and affecting the economies and livelihood of the South Asia populations.

## METHODS

We utilized publicly available COVID-19 data from the SAARC countries collected from January 2020 to 31 March 2021. Data was censured in March 2021, since countries in the SAARC region started reporting false results, as the COVID-19 pandemic resulted in massive deaths [12]. We gathered the countries’ data on COVID-19 incidence and mortality from the WHO COVID-19 dashboard [13] and used to determine the monthly case-fatality ratio (CFR).

Most countries (including Bangladesh, Bhutan, and Sri Lanka) had testing data recorded in the “Our World in Data” (OWD) database, where test-positivity was defined as the positive test percentage of all the COVID-19 tests conducted (i.e. true positive COVID-19 tests/all positive COVID-19 tests) [14]. We recorded India’s daily and weekly testing from its COVID-19 website [15], while we recorded the data for Nepal and Pakistan separately from documented COVID-19 situation reports [16,17]. We found non-OWD sources through common database websites [18].

However, some of the countries of interest had significant amounts of missing data. Observed testing for Afghanistan was only recorded from 10 February 2020 to 26 December 2020, for instance, while testing for the other SAARC countries was recorded from January 2020 through 31 March 2021. We primarily used OWD database for consistency in data retrieval, resorting to other sources if needed. For example, we used the Institute for Health Metrics and Evaluation (IHME) database for Afghanistan’s testing data [19].

We evaluated the quantitative health policy data using the COVID-19 Government Response Stringency Index from the OWD database [20]. It represents a composite score based on a set of health policy and response measures (ranging from school and workplace closures to limitations on public events, gatherings, and lockdowns). We obtained the community response to the governments’ health policies and enacted COVID-19 measures from IHME, which is calculated as the percentages of people “masking” in public and “social distancing” based on population mobility estimates from cell phone data. Because Bhutan is not included the IHME database, we were unable to collect any of its data.

Macroeconomic data on SAARC countries included economic growth (rate of change of real gross domestic product (GDP)), unemployment rate, consumer price index (CPI), and inflation. We collected these data from the repository Global Economy website [21] and the World Bank [22] to determine how the health policies and response measures impacted the economy and livelihood of populations during the COVID-19 pandemic.

### Statistical analysis

We performed population correction using the 2020 mid-year population standardized per 100 000 population for SAARC countries. We reported the categorical variables (case-fatality and test-positivity) as percentages.

We performed joinpoint regression analysis to test the statistical significance of changes in linear segments of temporal trends at joinpoints, utilizing the Monte Carlo permutation method to identify values that were weighted and averaged to determine the average monthly percent change (AMPC) and average weekly percent change (AMPC). The AMPC and AWPC provide single values for changes in temporal trends throughout the study period [23]. We considered a *P* < 0.05 as statistically significant. We determined the AWPC/AMPC's and their 95% confidence intervals (CI) across the SAARC countries for the following variables: the number of COVID-19 cases (weekly incidence per 100 000 population), number of deaths (weekly mortality per 100 000 population), monthly case-fatality (in percentage), COVID-19 testing (weekly testing per 100 000 population), weekly test positivity (in percentage), and weekly inflation and CPI. Since periodic data for unemployment and rate of change of real GDP was unavailable, we determined the percentage change from 2019 (before pandemic) to 2020 (most of the pandemic study period). After testing for normality using Shapiro-Wilk’s test, we used the Generalized extreme studentized deviate (ESD) test to identify outliers in unemployment and rate of change in the real GDP data set. We used the Joinpoint Regression Program version 4.8.0.1 (National Cancer Institute, Bethesda, MD, USA) for the jointpoint regression analyses [24].

## RESULTS

The COVID-19 health and response measures utilized by the SAARC countries are summarized in **Table 1**. In mid-2020, SAARC countries had a population of approximately 1 856 376 663 inhabitants, documenting 14 25 390 (767.67 per 100 000 population) COVID-19 cases and 194 698 deaths from January 2020 to 31 March 2021 (**Table 1**).

**Table 1:** COVID-19 epidemiological indicators from January 2020 to 31 March 2021

	Population (mid-year 2020)	COVID-19 cases (per 100 000 population)	COVID-19 deaths (per 100 000 population)	Case-fatality (%)	COVID-19 tested (per 100 000 population)	Test-positivity (%)
Afghanistan*	38 928 346	145.6	6.4	4.41%	894.6	16.28%
Bangladesh	164 689 383	382.7	5.6	1.46%	2835.7	13.50%
Bhutan	771 608	115.5	0.1	0.11%	75 787.7	0.15%
India	1 380 004 385	904.7	11.9	1.32%	17 738.9	5.10%
Maldives	540 544	4363	12.4	0.28%	-	-
Nepal	29 136 808	953.3	10.4	1.09%	7 812.5	12.20%
Pakistan	220 892 340	309.1	6.7	2.15%	4634.7	6.67%
Sri Lanka	21 413 249	433.3	2.7	0.61%	11 413.2	3.80%

*Observed testing for Afghanistan was only recorded from 10 February 2020 to 26 December 2020.

Each SAARC country invested early in rapid testing technology, hoping to achieve early detection. India and Sri Lanka both focused on limiting mobility, planning for rapid containment and quarantine and utilizing local police forces to arrest citizens not compliant with current mandates, hoping to be able to ease restrictions early on. Pakistan had a national plan with “smart localized lockdowns”. Bangladesh followed the WHO’s recommended response, creating a six-pillar plan and activating its army to ensure compliance. Nepal’s plan was not well defined, but the actions taken favoured increasing care for sick individuals, seeing early increases in hospital capacity and early tracing of known cases. Both the Maldives and Bhutan made an interesting choice by emphasizing economic support and stability.

**Figure 1:**
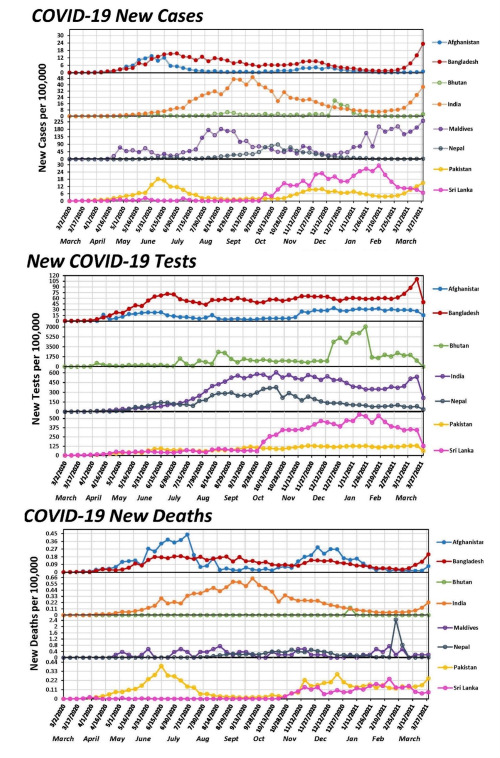
Incidence of COVID-19, deaths with COVID-19, number of COVID-19 tests, from January 2020 to March 2021.

**Figure 2:**
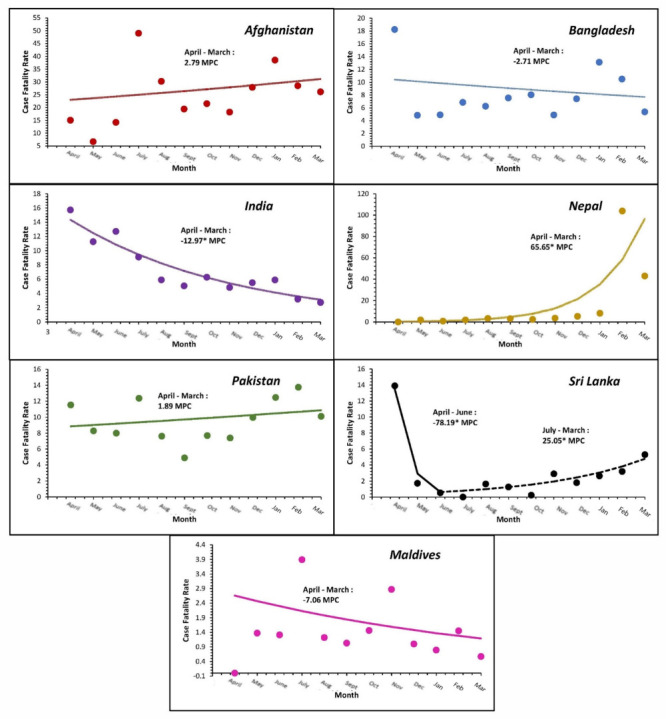
Average monthly percent changes in case fatality rates per country.

The COVID-19 cases, testing and mortality trends in South Asia are shown in **Figure 1**. We calculated and analysed monthly CFR for the period from April 2020 to March 2021 (**Figure 2**), excluding Bhutan because it only reported one death throughout this period. Only India, Nepal, and Sri Lanka showed statistically significant CFR monthly percent changes (MPCs). India saw a downward trend of CFR (MPC = -12.97; *P* < 0.05), while Sri Lanka initially saw a decrease in MPC between April and June 2020 (MPC = -78.19; *P* < 0.05) followed by an increase between July 2020 and March 2021 (MPC = 25.05, *P* < 0.05). Nepal saw a positive trend from April 2020 through March 2021 (MPC = 65.65; *P* < 0.05), primarily due to a large spike in CFR during February 2020 (**Figure 2**).

Bhutan performed the most COVID-19 tests per 100 000 persons (n = 75 787), while Afghanistan performed the least (n = 894). No testing information was available for the Maldives. Bhutan consistently had the most aggressive testing efforts throughout the study period, with a peak of 7041 per 100 000 during the last week of January 2021.

**Figure 3:**
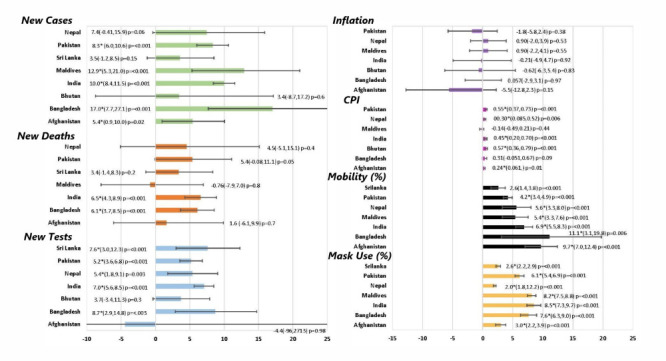
AWPC from joinpoint regression of COVID-19 indicators, with CPI displayed as the AMPC.

All countries had positive AWPC for new COVID-19 cases, but Bhutan’s, Sri Lanka’s, and Nepal’s AWPCs were not statistically significant. Of the significant AWPCs, Bangladesh’s was the highest (AWPC = 17.0; 95% CI = 7.7-27.1, *P* < 0.001), followed by Maldives (AWPC = 12.9; 95% CI = 5.3-21.0, *P* < 0.001) and India (AWPC = 10.0; 95% CI = 8.4-11.5, *P* < 0.001). (**Figure 3**). We analysed COVID-19 mortality data from 16 March 2020 to 31 March 2021 for all countries except Bhutan, which had one death. Only India (AWPC = 6.5; 95% CI = 4.3-8.9, *P* < 0.001) and Bangladesh (AWPC = 6.1; 95% CI = 3.7-8.5, *P* < 0.001) had statistically significant AWPCs. COVID-19 new tests administered AWPC were significantly positive in all countries except Afghanistan and Bhutan, with the latter most likely resulting from the country’s aggressive and consistent COVID-19 testing. Bangladesh had the largest significant positive AWPC of new tests administered (AWPC = 8.7; 95% CI = 2.9-14.8, *P* = 0.003), followed by Sri Lanka (AWPC = 7.6; 95% CI = 3.0-12.3, *P* < 0.001) and India (AWPC = 7.0; 95% CI = 5.6-8.5, *P* < 0.001).

**Figure 4:**
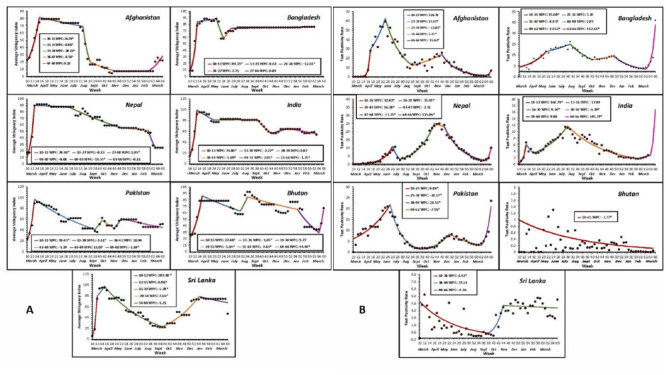
AWPC from joinpoint regression. **Panel A.** Government response stringency index. **Panel B.** Test positivity per country.

The Government Response Stringency Index joinpoint regression AWPCs reflecting the government health policy and restrictions showed a statistically significant sharp spike uniformly across the SAARC from March to April 2020 (**Figure 4**). Following the initial spike, Afghanistan (-0.86), Bangladesh (0.63), and Nepal (-0.13) had a relative plateau in the index, as did India (0.07) after a small dip. Afghanistan had a sharp decline in the index between August and September, after which the health policy restrictions continued at the lowest trend among all SAARC countries. India and Nepal had similar pattern of a plateau and a gradual decline. Bangladesh had a plateau at the highest level of index among SAARC countries from June to March 2021, while Pakistan, Bhutan, and Sri Lanka had a somewhat similar see-saw pattern, with a sharp decline in the index followed by an increase in the government health policy restrictions (**Figure 4**).

Each country saw a dramatic decline in the IHME mobility composite between February and April 2020, except for Bhutan, which had no available mobility data. Sri Lanka saw the greatest decrease with a trough composite of -85. Simultaneously, Afghanistan saw the smallest decline with a trough of -44. We calculated AWPC data from mid-April 2020 to March 2021 to examine the changes in mobility after these early dramatic declines. All countries had a statistically significant positive AWPC, with Bangladesh the greatest (AWPC = 11.1; 95% CI = 3.1-19.8, *P* = 0.006) and Sri Lanka the smallest increase in mobility (AWPC = 2.6; 95% CI = 1.4-3.8, *P* < 0.001). Mask usage data was analyzed from the beginning of March 2020 through March 2021, except for Bhutan, which lacked available data. All countries had a statistically significant positive AWPC. India had the greatest mask usage (AWPC = 8.5; 95% CI = 7.3-9.7, *P* < 0.001), while Nepal had the smallest (AWPC = 2.0; 95% CI = 1.8-12.2, *P* < 0.001).

The AMPC for inflation was insignificant across all SAARC countries, while the CPI had a statistically significant increase for Bhutan (0.57), Pakistan (0.55), India (0.45), Nepal (0.3), and Afghanistan (0.24). Periodic unemployment data for the joinpoint regression analysis was unavailable for all SAARC countries except India. Among the SAARC countries, Bhutan had the most significant increase in unemployment (64.76%), followed by Nepal (55.79%) and India (34.91%), while Afghanistan (6.83%), Pakistan (16.83%), and Sri Lanka (13.35%) had the lowest increase in unemployment. We also analysed the percentage change in the rate of change of real GDP; Maldives had the highest decrease (557.51%), followed by India (297.03%), and Sri Lanka (257.97%), while Pakistan (46.46%) and Bangladesh (70.80%) had the lowest decrease (**Table 2**).

**Table 2:** Total unemployment (% of total labour force) and rate of change of real GDP in SAARC countries

	**Unemployment**			**Rate of change of real GDP**		
**Country**	**2019**	**2020**	**Change**	**2019**	**2020**	**Change**
Afghanistan	10.98	11.73	+6.83%	3.91	-1.93	-149.36%
Bangladesh	4.22	5.30	+25.59%	8.15	2.38	-70.80%
Bhutan	2.27	3.74	+64.76%	5.46	-6.77	-223.99%
India	5.27	7.11	+34.91%	4.04	-7.96	-297.03%
Sri Lanka	4.27	4.84	+13.35%	2.26	-3.57	-257.97%
Maldives	5.82	7.18	+23.36%	6.99	-31.98	-557.51%
Nepal	2.85	4.44	+55.79%	6.66	-2.09	-131.38%
Pakistan	3.98	4.65	+16.83%	0.99	0.53	-46.46%

GDP – gross domestic product, SAARC – South Asian Association for Regional Cooperation

## DISCUSSION

To the best of our knowledge, this is the first study to determine the impact and implications the COVID-19 pandemic has had on the SAARC region with an approximate population of nearly two billion. The study analysed health policies, epidemiology indicators, and public health capacity while considering macroeconomic influences and outcomes.

Among the SAARC nations, the Maldives had the highest number of COVID-19 cases and deaths per 100 000 population and Bhutan had the lowest. Our findings for the Maldives, albeit seemingly disproportionate in comparison to other SAARC countries), are in line with other literature [25]. We demonstrated that Afghanistan (4.41%) and Pakistan (2.15%) had the highest case-fatality percentage, in line with other literature [25]. Finally, although our study showed that Bhutan (n = 75 787.7) performed the most COVID-19 tests per 100 000 people, followed by India (n = 17 738.9), other studies placed India first, followed by the Maldives and Bhutan [25]. The discrepancy likely derives from the other study’s analysis of data ranging from 1 February to 1 September 2020, while our study continued until March 2021. In our study period, countries with smaller populations such as the Maldives and Bhutan ramped up COVID-19 testing capabilities. In line with our findings, all SAARC countries demonstrated decreased mobility around April 2020, with each showing variability returning to baseline mobility [26]. SAARC countries may have avoided the higher case fatality rates seen in Europe and North America due to a younger average age (24.4 years) of their population. Furthermore, the pandemic reached South Asia later, so SAARC countries were able to implement earlier lockdowns [25].

The SAARC region closed its borders to international travel early on; each country restricted air flight in early March and nearly all governments established a complete lockdown or significantly restricted public movement by the summer of 2020, as is also shown by the Government Response Stringency Index (**Figure 4**). All SAARC countries had started administering COVID-19 vaccines by March 2021 to their populations. After the initial health policy restrictions, Sri Lanka had the largest drop in COVID-19 health policy restrictions from April to September 2020 (AWPC = -5.28; *P* < 0.05), which resulted in a rise in COVID-19 cases and deaths; the government rapidly responded with tightening health policy from late September 2020 to January 2021. The Indian government’s plan of “test-track-treat” and its implementation of national/state-wide lockdown from late March 2020 was extended to May 2020 and was followed by a near static government response (AWPC = 0.07; *P* > 0.05) until September 2020, after which the national health policy and response measures loosened further. Simultaneously, partial lockdowns were introduced in designated containment zones with high infection rates, where the populations were quarantined and had restricted movements [25,27]. Other countries with periods of national lockdowns and similar static government responses included: Nepal from April 2020 to June 2020 (WPC = -0.13; *P* > 0.05) and November 2020 to January 2021 (WPC = -0.68; *P* > 0.05). Bangladesh also had a lockdown and static government health policy response (WPC = 0.09; *P* > 0.05) from July 2020 onwards, introducing containment zones and lockdowns in red-zone designated areas with high COVID-19 infection rates and less restrictions in green and yellow zones with lower infection rates. By August 2020, however, the rate of new cases began to decrease throughout the region, except in India, Nepal, and the Maldives, where COVID-19 cases continued, as per our findings (for all three countries) and previous studies (for Nepal and India) [28,29]. When comparing the approach of India, Nepal, and Bangladesh to Bhutan, Pakistan, and Sri Lanka (to a certain extent), where the governmental approach was proactive in a seesaw fashion (with sharp bursts of increased restrictions followed by gradual decreases resulting in a stable and decreased rate of new cases), the assumption that maintaining health policy restrictions in short bursts beyond the initial decline in cases was instrumental in containment [30,31] (**Figure 4**).

The health policy vs economy tradeoff in the ongoing COVID-19 pandemic has grave implications for developing countries. Cessation of economic activity resulting from harsh COVID-19 health response and policy measures like national and state-wide lockdowns and border controls has resulted in massive unemployment and pushed millions of people into poverty, acute hunger, and food insecurity [32,33]. SAARC countries (Pakistan, Nepal, and a few regions in India) were included at the highest risk for an economic shock during the COVID-19 pandemic [34]. The macroeconomic results correlate with the shifts in COVID-19 epidemiologic data; increased unemployment, near static trends in CPI and insignificant results for inflation, decreasing current account balance, and GDP growth from decreased exports due to isolation and destabilization to global supply chain networks. Each country saw expected increases in unemployment (**Table 2**). Bhutan had the largest percentage increase in the unemployment rate despite the successful health strategy that resulted in the lowest number of COVID-19 cases and deaths. This result is explained approximately by one-third loss of jobs in Bhutan’s tourism sector due to the pandemic [35]. The Maldives, also heavily dependent on tourism, had the highest decrease in the rate of percentage change of real GDP which correlates with the highest number of COVID-19 cases and mortality (per 100 000 population).

Nepal had the second-highest percentage increase in the unemployment rate, which correlates with the highest number of COVID-19 cases and the third-highest COVID-19 mortality. India had the third-highest percentage increase in the unemployment rate and second-highest decrease in the rate of percentage change of real GDP which correlates with the second-highest COVID-19 disease burden among SAARC countries. A recent study also described a similarly rapid rise in the unemployment trend in India; urban employment decreased by 31% between March and April 2020 [36]. Nepal, with a much smaller population than India, also showed a similar pattern. However, Pakistan had the third-lowest percentage increase in unemployment and the lowest decrease in rate of percentage change of real GDP which correlated with a relatively lower COVID-19 disease burden (**Figure 4**). The key to Pakistan’s successful health policy and economic outcomes were targeted, time-limited, and localized lockdowns with health policy stringency index demonstrating a rapid seesaw pattern that approximated the test-positivity joinpoint pattern (**Figure 4**). In the case of a large number of tests like Bhutan, the test-positivity trend might not be an accurate indicator and disease incidence should be used to approximate health policy stringency.

The biggest limitation of our study is the direct comparison of data from diverse countries with huge differences in the size of populations, land mass, and economies. Other limitations included differences in reporting methods, quality, and definitions used by governments and organizations when reporting data to COVID-19 databases. The SAARC countries included in the study comprise a diverse mix of large and small populations that we directly pooled and compared. In some cases, the data was reported as best estimates. As all COVID-19 data currently is “as reported,” the actual value is likely to be much higher. The epidemiology and outcomes data are also likely to be influenced by other factors for which data was not collected, including hospitalizations, accessibility and quality of diagnostics and health care, and public’s perception towards testing. Some macroeconomic indicators have not been reported recently or at all, so we could not use them in this evaluation. As they are related to changes in the economy or specific changes in health policy, we did not analyse or control for confounding factors. There have been efforts in the field of diagnostic machine learning that may also play a role in how these countries implement different versions of the lockdowns [37].

## CONCLUSIONS

The few studies that have investigated the trade-off between health policy and economy during the COVID-19 pandemic have reported that it does not exist [38,39]. However, we found that there was a trade-off between strict and protracted health policy and response measures and the economies in the SAARC region during the COVID-19 pandemic. Developing low-income and low-middle-income countries, with extended periods of strict health policies in the absence of social welfare programs, were relatively ineffective in controlling COVID-19 transmission, which resulted in unemployment and adversely affected the economy. The loss of jobs in urban centres led to mass migrations of migrant city workers back to their villages in the face of extended national or state-wide lockdowns, which also led to viral transmission and may have been counterproductive [39].

For developing countries, the health policy and response measures in a pandemic need to be proactive, targeted, and time-limited in a rapid seesaw manner that should approximate the test-positivity pattern or incidence, as was demonstrated by Pakistan and (to some extent) Sri Lanka (**Figure 4**). Such a pandemic response and health policy would break the spread of the pandemic while limiting cessation of economic activity to prevent mass unemployment and acute food insecurity in the short-term.

## References

[1] World Bank. Prospects of an economic rebound in South Asia are firming up as growth is set to increase by 7.1 percent in 2021 and in 2022, but growth is uneven and economic activity well below pre-COVID-19 estimates. 2021. Available: https://www.worldbank.org/en/region/sar/overview#1. Accessed: 11 April 2023.

[2] Editors of Encyclopaedia Britannica. South Asian Association for Regional Co-operation. 2017. Available: https://www.britannica.com/topic/South-Asian-Association-for-Regional-Co-operation. Accessed: 11 April 2023.

[3] The Nuclear Threat Initiative. South Asian Association for Regional Cooperation (SAARC). 2007. Available: https://www.nti.org/education-center/treaties-and-regimes/south-asian-association-regional-cooperation-saarc/. Accessed: 11 April 2023.

[4] World Health Organization. WHO Director-General's opening remarks at the media briefing on COVID-19 - 11 March 2020. 2020. Available: https://www.who.int/director-general/speeches/detail/who-director-general-s-opening-remarks-at-the-media-briefing-on-covid-19—11-march-2020. Accessed: 11 April 2023.

[5] Khan IM Khan S Chotani R Laaser U SARS and SAARC: lessons for preparedness. J Ayub Med Coll Abbottabad. 2003;15:1-2. 14552237

[6] Phua J Faruq MO Kulkarni AP Redjeki IS Detleuxay K Mendsaikhan N Critical Care Bed Capacity in Asian Countries and Regions. Crit Care Med. 2020;48:654-62. 10.1097/CCM.0000000000004222 31923030

[7] South Asian Association of Regional Cooperation. COVID19 Emergency Fund. 2022. Available: http://covid19-sdmc.org/covid19-emergency-fund. Accessed: 11 April 2023.

[8] Babu GR Khetrapal S John DA Deepa R Narayan KMV Pandemic preparedness and response to COVID-19 in South Asian countries. Int J Infect Dis. 2021;104:169-74. 10.1016/j.ijid.2020.12.048 33370566 PMC7836380

[9] Deyshappriya R. Examining poverty trends in South Asian countries: where is Sri Lanka among its South Asian counterparts? 2018. Available: https://blogs.lse.ac.uk/southasia/2018/07/31/examining-poverty-trends-in-south-asian-countries-where-is-sri-lanka-among-its-south-asian-counterparts/. Accessed: 11 April 2023.

[10] Timmer H, Mercer-Blackman V, Beyer RC. The economic impact of COVID-19 on South Asia: 3 Visuals. 2020. Available: https://blogs.worldbank.org/endpovertyinsouthasia/economic-impact-covid-19-south-asia-3-visuals. Accessed: 11 April 2023.

[11] Atul A. Analysis | South Asia unveils India-China balancing act during COVID-19. 2020. Available: https://www.thehindu.com/news/national/analysis-south-asia-unveils-india-china-balancing-act-during-covid-19/article31524571.ece. Accessed: 11 April 2023.

[12] Biswas RK Afiaz A Huq S Underreporting COVID-19: the curious case of the Indian subcontinent. Epidemiol Infect. 2020;148:e207. 10.1017/S0950268820002095 32912354 PMC7508490

[13] World Health Organization. WHO Coronavirus (COVID-19) Dashboard. 2022. Available: https://covid19.who.int/. Accessed: 11 April 2023.

[14] Ritchie H, Mathieu E, Rodés-Guirao L, Appel C, Giattino C, Ortiz-Ospina E, et al. Coronavirus Pandemic (COVID-19). Available: https://ourworldindata.org/coronavirus. Accessed: 11 April 2023.

[15] COVID19India. Coronavirus Outbreak in India - covid19india.org. 2020. Available: https://www.covid19india.org/. Accessed: 14 January 2022.

[16] Ministry of Health and Population Nepal. CoVid19-Dashboard. 2022. Available: https://covid19.mohp.gov.np/situation-report. Accessed: 14 January 2022.

[17] Ministry of National Health Services Regulations and Coordination. COVID-19 Health Advisory Platform by Ministry of National Health. 2022. Available: https://www.covid.gov.pk/. Accessed: 14 January 2022.

[18] Worldometer. COVID-19 coronavirus pandemic. 2022. Available: https://www.worldometers.info/coronavirus/. Accessed: 23 April 2023.

[19] Institute for Health Metrics and Evaluation. COVID-19 Projections. 2022. Available: https://covid19.healthdata.org/global?view=cumulative-deaths&tab=trend. Accessed: 14 January 2022.

[20] Our World in Data. COVID-19 Stringency Index, Jan 13, 2022. 2020. Available: https://ourworldindata.org/grapher/covid-stringency-index. Accessed: 11 April 2023.

[21] Global Economy. Global economy, world economy | TheGlobalEconomy.com. Available: https://www.theglobaleconomy.com/. Accessed: 14 January 2022.

[22] World Bank. Unemployment, total (% of total labor force) (modeled ILO estimate). Available: https://data.worldbank.org/. Accessed: 14 January 2022.

[23] Kim HJ Fay MP Feuer EJ Midthune DN Permutation tests for joinpoint regression with applications to cancer rates. Stat Med. 2000;19:335-51. 10.1002/(SICI)1097-0258(20000215)19:3<335::AID-SIM336>3.0.CO;2-Z 10649300

[24] National Cancer Institute. Joinpoint Trend Analysis Software. Available: https://surveillance.cancer.gov/joinpoint/. Accessed: 30 March 2023.

[25] Babu GR Khetrapal S John DA Deepa R Narayan KMV Pandemic preparedness and response to COVID-19 in South Asian countries. Int J Infect Dis. 2021;104:169-74. 10.1016/j.ijid.2020.12.048 33370566 PMC7836380

[26] Shohan MUS Ul Alam ASMR Ralkhi NN Kabir M Siam MKS Hasan MM Onset, Transmission, Impact, and Management of COVID-19 Epidemic at Early Stage in SAARC Countries. Authorea. 2020. 10.22541/au.159775079.90952648

[27] Bhattacharjee A Kumar M Patel KK When COVID-19 will decline in India? Prediction by combination of recovery and case load rate. Clin Epidemiol Glob Health. 2021;9:17-20. 10.1016/j.cegh.2020.06.004 32838057 PMC7308770

[28] Yang W Shaman J COVID-19 pandemic dynamics in India and impact of the SARS-CoV-2 Delta (B.1.617.2) variant. medRxiv. 2021. 10.1098/rsif.2021.0900 PMC9169547 35670221

[29] Pantha B Acharya S Joshi HR Vaidya NK Inter-provincial disparity of COVID-19 transmission and control in Nepal. Sci Rep. 2021;11:13363. 10.1038/s41598-021-92253-5 34172764 PMC8233407

[30] Jung J A Long Way to the Recovery: COVID-19 Will Not Disappear. J Korean Med Sci. 2021;36:e231. 10.3346/jkms.2021.36.e231 34402229 PMC8369311

[31] Makhoul M Chemaitelly H Ayoub HH Seedat S Abu-Raddad LJ Epidemiological Differences in the Impact of COVID-19 Vaccination in the United States and China. Vaccines (Basel). 2021;9:223. 10.3390/vaccines9030223 33807647 PMC8002114

[32] Townsend RF, Gautam M. Responding to a stark rise in food insecurity across the poorest countries. 2021. Available: https://blogs.worldbank.org/voices/responding-stark-rise-food-insecurity-across-poorest-countries. Accessed: 11 April 2023.

[33] Haas A, Khan A, Khwaja A. Policymaking in uncertain times: Smart containment with active learning. 2020. Available: https://www.theigc.org/wp-content/uploads/2020/05/Haas-et-al-2020-brief_final.pdf. Accessed: 9 October 2020.

[34] Djankov S, Pannizza U. Vulnerable Solidarities: Identity, Spatiality and the Contentious Politics of Migration. 2020. Geneva: Graduate Institute Publications; 2020.

[35] Lhawang U. Moving on from the pandemic in Bhutan. 2021. Available: https://www.policyforum.net/moving-on-from-the-pandemic-in-bhutan/. Accessed: 11 April 2023.

[36] Mamgain RP Understanding labour market disruptions and job losses amidst COVID-19. J Soc Econ Dev. 2021;23:1-19. 10.1007/s40847-020-00125-x 34720484 PMC7903218

[37] Naseem M Arshad H Hashmi SA Irfan F Ahmed FS Predicting mortality in SARS-COV-2 (COVID-19) positive patients in the inpatient setting using a novel deep neural network. Int J Med Inform. 2021;154:104556. 10.1016/j.ijmedinf.2021.104556 34455118 PMC8378987

[38] Kaplan S Lefler J Zilberman D The political economy of COVID-19. Appl Econ Perspect Policy. 2022;44:477-88. 10.1002/aepp.13164 34230850 PMC8250203

[39] Irfan FB Minetti R Telford B Ahmed FS Syed AY Hollon N Coronavirus pandemic in the Nordic countries: Health policy and economy trade-off. J Glob Health. 2022;12:05017. 10.7189/jogh.12.05017 35932219 PMC9356530

[40] Rajalakshmi TK. Centre blames media “fake news” for mass migration during lockdown. 2020. Available: https://frontline.thehindu.com/dispatches/article31228357.ece. Accessed: 11 April 2023.

